# Modeling Gastrulation in the Chick Embryo: Formation of the Primitive Streak

**DOI:** 10.1371/journal.pone.0010571

**Published:** 2010-05-11

**Authors:** Bakhtier Vasiev, Ariel Balter, Mark Chaplain, James A. Glazier, Cornelis J. Weijer

**Affiliations:** 1 Division of Mathematics, University of Dundee, Dundee, United Kingdom; 2 Biocomplexity Institute and Department of Physics, Indiana University, Bloomington, Indiana, United States of America; 3 Wellcome Trust Biocentre, School of Life Sciences, University of Dundee, Dundee, United Kingdom; University of Nottingham, United Kingdom

## Abstract

The body plan of all higher organisms develops during gastrulation. Gastrulation results from the integration of cell proliferation, differentiation and migration of thousands of cells. In the chick embryo gastrulation starts with the formation of the *primitive streak*, the site of invagination of mesoderm and endoderm cells, from cells overlaying Koller's Sickle. Streak formation is associated with large-scale cell flows that carry the mesoderm cells overlying Koller's sickle into the central midline region of the embryo. We use multi-cell computer simulations to investigate possible mechanisms underlying the formation of the primitive streak in the chick embryo. Our simulations suggest that the formation of the primitive streak employs chemotactic movement of a subpopulation of streak cells, as well as differential adhesion between the mesoderm cells and the other cells in the epiblast. Both chemo-attraction and chemo-repulsion between various combinations of cell types can create a streak. However, only one combination successfully reproduces experimental observations of the manner in which two streaks in the same embryo interact. This finding supports a mechanism in which streak tip cells produce a diffusible morphogen which repels cells in the surrounding epiblast. On the other hand, chemotactic interaction alone does not reproduce the experimental observation that the large-scale vortical cell flows develop simultaneously with streak initiation. In our model the formation of large scale cell flows requires an additional mechanism that coordinates and aligns the motion of neighboring cells.

## Introduction

Gastrulation is a critical stage in the development of all higher organisms, since it is the stage where the three germ layers, the ectoderm, mesoderm and endoderm, assume their definitive positions in the embryo [1]. Cells proliferate, differentiate and migrate extensively during gastrulation. The chick embryo is a convenient model organism for investigation of amniote gastrulation, since it is essentially flat, transparent and develops outside the mother. Cell movement during gastrulation is similar in avians and humans. At the time of egg laying, the chick embryo consists of around twenty to thirty thousand cells. A subset of these cells forms a one-cell-layer thick, quasi-epithelial disk, the *epiblast*. At the periphery of the embryo, the epiblast sits on top of a rigid, several-cell-thick layer of large mesenchymal cells, which directly contact the underlying yolk. This outer segment of the embryo is known as the *Area Opaca* (*AO*). In the central part of the embryo, the *Area Pellucida* (*AP*), clusters of a few small rounded cells attach to the ventral (bottom) side of the epiblast, forming the *primary hypoblast*. During the initial course of development, the primary hypoblast flattens to form an epithelial layer of large thin cells, the *hypoblast*. The AP epiblast cells give rise to the embryo proper, while the hypoblast and AO form extra-embryonic structures. The band of epithelial cells at the posterior lateral boundary between the AO and the AP has an elongated shape, forming the *marginal zone*.

**Figure 1:**
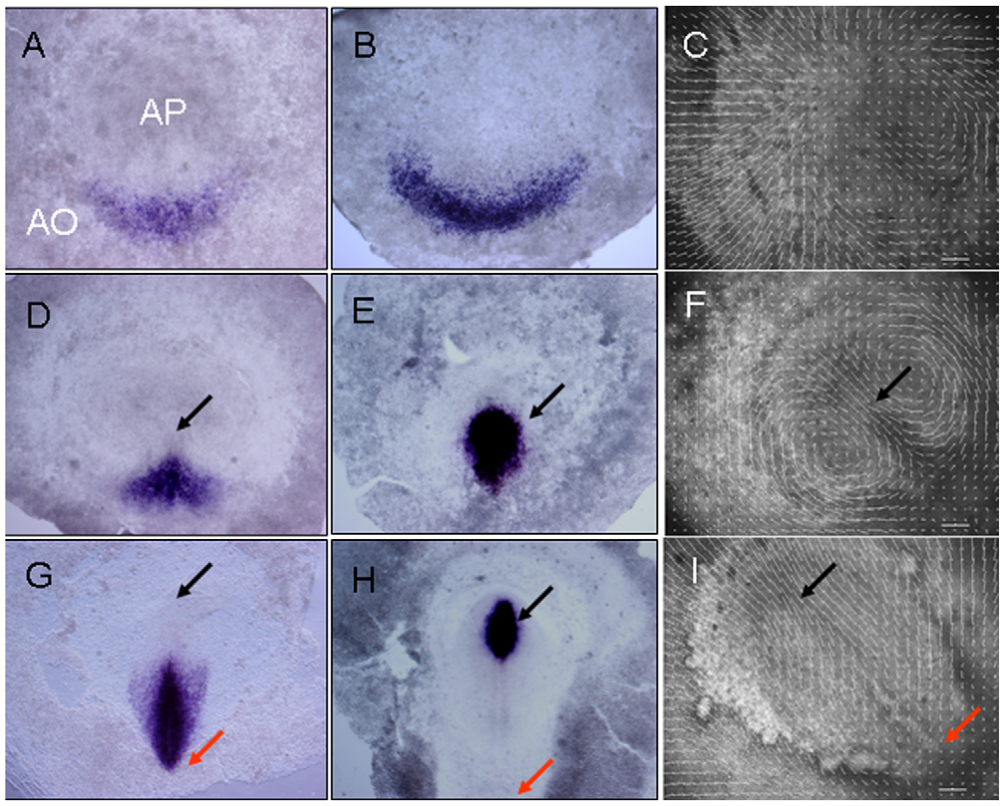
Critical stages of the development of the primitive streak in the chick embryo. Wnt 8c expression during formation of Koller's sickle and the primitive streak. (**A, B**) At stage HH1 [39] Wnt8c (**A**) and Chordin (**B**) RNAs are expressed in the *Area Opaca*. At this time the embryo is growing and the epiblast increases in size as shown by the outward-pointing velocity arrows (**C**). At stage HH2, Wnt8c (**D**) and Chordin (**E**) RNA are both expressed along the primitive streak, but the cell flow patterns have changed to two counter-rotating cells flows (**F**). At stage HH4, Wnt8c (**G**) is expressed in the base of the streak, while Chordin RNA is expressing in the tip of the streak and the surrounding forming neural plate (**H**). During this phase the large-scale rotational movements start to transform into flows along the anterior-posterior axis of elongation of the embryo (*I*). AO *Area Opaca*, AP *Area Pellucida*, black arrow tip of the streak, red arrow base of the streak. In the velocity flow fields the thick horizontal white bar indicates cell flow speeds of *1* µm/min. Images C, F and I were taken at *t* = 0 minutes, *t* = 300 minutes and *t* = 800 minutes from the start of the experiment at HH1. See Supplementary Materials, movie S1.

Initially, the embryo appears circularly symmetric. Then, a group of deep mesenchymal cells at the boundary between the AO and AP in the posterior half of the embryo thicken to form *Koller's Sickle*, a darker sickle-shaped or lunate region. Inductive signals from the marginal zone, an anterior-posterior gradient of Vg1 and graded Wnt8c expression in the AO induce nodal expression in the epiblast overlying Koller's Sickle and the nodal-expressing cells then differentiate to form mesendoderm (Fig. 1A) [2], initiating gastrulation. Gastrulation starts with the formation of the *primitive streak* (*PS*), as mesendoderm from the sickle-shaped region at the interface between the AO and AP moves into the posterior midline region of the embryo (Fig. 1). The development of the freshly laid egg (stage EG X) to the formation of a fully extended streak (stage HH4) takes roughly 24 hours. Streak formation is concurrent with large vortical flows of cells in the epiblast (Fig. 1). These vortices rotate in opposite directions—away from the midline in the anterior and towards the midline in the posterior [3], [4], [5], [6], [7]. In the posterior of the epiblast, where cell flows meet, the cells start to stack on top of each other and the epiblast becomes several cell-diameters thick, forming the structure visible as the streak (HH1-2). The streak extends progressively in the anterior direction until it reaches ∼80% of the length of the AO (HH4) (Supplementary Materials Movie S1).

Preceding streak formation, the *secondary hypoblast* forms in the posterior to anterior direction by flattening of the clusters of cells of the primary hypoblast to form an epithelial sheet, incorporating cells migrating out from the deeper layers of Koller's sickle in the anterior direction (Stage EG XIII). After the streak has extended halfway across the epiblast, the deeper cells of the streak start to move radially away from the midline as they are replaced by epiblast cells that ingress into the streak after undergoing an epithelial-to-mesenchymal transition. The movement pattern of the cells in the epiblast then changes and cells in the lateral epiblast start to move medially towards the streak to replace the cells that the streak loses to ingression. The first cells to ingress form the *endoblast* or *definitive endoderm*, which replaces the secondary hypoblast, and movement of these cells mirrors the movement of cells in the epiblast during the formation of the streak (Stage HH3-HH3+) [8]. The formation and extension of the visible primitive streak (HH1-HH8) takes around 12 hours. Throughout this period, cells in the AP divide at a low rate and a small percentage of them ingress directly, without joining the primitive streak [9]. Unless we specify otherwise, we do not consider the effects of cell division or ingression in this paper.

Cells in the anterior part of the streak express different mesodermal marker genes from cells in the posterior part of the streak. Genes expressed in the anterior streak include inhibitors of the BMP and Wnt signaling pathways, members of which are expressed in the posterior streak (Fig. 1). *Hensen's node* is formed from a small group of streak cells located at the tip of the fully-extended primitive streak and is recognizable as a distinct morphological structure. It plays a key role in the induction of the nervous system and acts as an organizer of cell movement during later gastrulation, especially during the streak regression stages in which the node moves posteriorly and the streak shortens until it finally disappears in the tailbud/somitogenesis stages. BMP inhibitors such as Chordin as well as Wnts such as Wnt8c are initially expressed in the sickle-shaped mesoderm. However, during streak formation, the expression of Chordin becomes restricted to the cells in the anterior streak, while Wnt8c is restricted to the posterior streak (Fig. 1). We do not know whether the cells that will form the tip of the streak initially intermingle with the cells that will form the posterior part of the streak and then sort out during streak extension, or whether the streak zones arise through differential reprogramming of gene expression *in situ*.

Gastrulation is complex, and its basic cellular and molecular mechanisms are still poorly understood. *E.g.*, ingressing mesoderm cells are essentially mesenchymal and move as individuals, but do so in a highly coordinated manner, forming frequent contacts with neighbors. The cell flows associated with streak formation take place in an epithelial layer of cells sitting on a complex basal lamina and connected by well developed apical adherens and tight junctions. However cells in the epiblast also move actively (autonomously) and must use some chemical or mechanical cues to coordinate their movements [4].

Cellular intercalation and chemotaxis have both been proposed as mechanisms directing cell migration during gastrulation [4], [6], [10], [11]. Recent data and theoretical considerations rule out the older hypothesis that cell migration during gastrulation results from localized and/or oriented cell division [4], [12]. New experimental data [7] show that a posterior domain in the forming streak contains polarized cells which appear to intercalate, at least on a limited scale. However, by itself, intercalation is a result of more specific cell behaviors rather than a cell-level mechanism. Intercalation could result from cells polarizing their protrusive activity, aligning themselves and pulling on each other, resulting in cell interdigitation and shortening the tissue in the direction of cell movement while lengthening it in the perpendicular direction. Such in-plane cell polarization can result from signaling through the *planar cell polarity pathway* (*PCP*). The non-canonical Wnt signaling pathway is one of several regulators of the PCP. Some experiments have found that inhibition of the Wnt-PCP pathway disturbs the formation of the primitive streak [7], but our experiments found that inhibition of this pathway had no major effect on streak formation [11]. Observed intercalation could also result from *chemotaxis* – the polarization of cells in response to a chemotactic signal, which produces directed migration through the localized activation of the actin-myosin cytoskeleton along the chemical gradient [13], [14]. Experimental evidence suggests that chemotactic agents guide cell migration during gastrulation [15]. Theoretical considerations also suggest that PS formation may involve chemotactic movement of cells in the epiblast [16].

In this paper we use computational methods to investigate the hypothesis that chemotactic movement of cells and differential adhesion suffice to explain formation of the primitive streak, and that chemotactic response together with differential adhesion and cell-cell induced polarization, suffice to reproduce the patterns of cell migration observed during gastrulation. Computer simulation can be an efficient tool to explore possible mechanisms and to design experiments to validate these mechanisms. In this paper we use a multi-cell simulation method (the Glazier-Graner Hogeweg model, the *GGHM*, aka the Cellular Potts model or *CPM*) originally developed by Glazier and Graner [17], [18], that has successfully been used to simulate *Dictyostelium discoideum* morphogenesis, blood vessel formation and somitogenesis in the chick embryo, and which suits our investigation of cellular mechanisms in gastrulation [19], [20], [21]. This methodology represents cells as spatially-extended collections of grid points (*voxels*) on a regular lattice. Concentration fields are stored in separate, parallel lattices. Updating the cell lattice according to specific rules allows simulation of cell growth, division, polarization, motion and differentiation, as well as cells' secretion and response to concentration fields. The concentration fields of extracellular signaling molecules obey partial-differential equations (*PDE*s) describing diffusion and decay (Eq. 10). Here, we simulate different combinations of cell behaviors to see whether they suffice to generate the in-plane cell movement patterns of gastrulation. Some combinations of behaviors reproduce patterns observed in biological experiments, while others do not (See Table 1).

**Table 1:** Summary of simulation results for different mechanisms.

Biological Mechanisms						Simulation Behaviors					
Non-Oriented Cell Division	Secretion	Chemotaxis	Chemotactic Mechanism	Induced Polarization	Differential Adhesion	Koller's sickle accretes to midline	Streak tip stays attached to Primitive Streak	Streak Quality	Vortices	Speed of Vortex Formation	Double-Streak Interaction
N	N	N		N	N	N	N	None	None	None	–
N	N	N		N	Y	**Y**	**Y**	None	None	None	–
N	S	−ST	M3	N	Y	**Y**	P	Poor	Weak	Late	–
N	ST	+S	M4	N	Y	**Y**	**Y**	Poor	Weak	Late	–
N	ST	−AP	M2	N	Y	**Y**	**Y**	**Good**	Weak	Late	–
N	AP	+ST	M1	N	Y	P	N	Poor	Weak	Late	–
Y	S	−ST	M3	N	Y	**Y**	N	Poor	Weak	Late	–
Y	ST	+S	M4	N	Y	**Y**	**Y**	Poor	Weak	Late	–
Y	ST	−AP	M2	N	Y	**Y**	**Y**	Poor	Weak	Late	–
Y	AP	+ST	M1	N	Y	P	P	Poor	Weak	Late	–
*N*	*S*	*−ST*	*M3*	*Y*	*Y*	***Y***	***Y***	***Good***	***Strong***	***Rapid***	***Repel***
N	ST	+S	M4	Y	Y	**Y**	**Y**	Poor	**Strong**	**Rapid**	None
N	ST	−AP	M2	Y	Y	**Y**	**Y**	**Good**	**Strong**	**Rapid**	Attract
N	AP	+ST	M1	Y	Y	**Y**	**Y**	**Good**	**Strong**	**Rapid**	None
Y	S	−ST	M3	Y	Y	**Y**	N	None	Weak	**Rapid**	–
Y	ST	+S	M4	Y	Y	**Y**	**Y**	**Good**	Weak	**Rapid**	–
Y	ST	−AP	M2	Y	Y	**Y**	**Y**	**Good**	Weak	**Rapid**	–
Y	AP	+ST	M1	Y	Y	**Y**	**Y**	**Good**	Weak	**Rapid**	–

We considered the roles of four mechanisms: differential adhesion, chemotactic signaling (Mechanisms M1–M4), induced polarization, and cell proliferation. In chemotactic signaling, one cell type secretes a signal and another cell type responds chemotactically. A “+” indicates attraction, and a “−” indicates repulsion. To assess our simulations we looked for four significant morphogenetic patterns observed *in vitro*: accretion of Koller's sickle cells to the midline, streak formation, the streak tip remaining attached to the streak during extension, and vortical motion in the AP (at the same time or just prior to streak extension). We indicate by yes (“Y”), no (“N”), or partial (“P”) whether these patterns are observed in simulations including the indicated mechanisms. **Streak Quality** was assessed subjectively as “None,” “Poor” or “Good” to indicate the similarity between simulated and experimental streak aspect ratios and the shape and location of the streak tip (Figs. 4, 5, 7). “None” indicates that no complete streak formed, “Poor” indicates a short, excessively broad streak, an excessively narrow streak, a streak with a split tip, or a streak where the streak nearly separated from the streak tip. “Good indicates that a streak formed where the tip remained connected, stayed well attached to the streak and the streak had an aspect ratio between 1∶5 and 1∶10. **Vortices** are assessed subjectively as “None,” “Weak” or “Strong” to indicate the size of the vortex. “None” indicates that the vortex filled less than 10% of the AP, “Weak” between 10% and 25% and “Strong” more than 25%. **Double Streak Interaction** summarizes the results in Fig. 6. **Speed of Vortex Formation** indicates whether the vortices form immediately (“rapid”) when streak progression begins or develop slowly as the streak progresses (“late”). **Bold** in the Table indicates experimentally observed results. “–” indicates no data. The *italics* in the Table indicate the simulation mechanisms which best agree with experiment.

Our simulations show that differential chemotaxis and cell-cell adhesion suffice to explain the formation of the primitive streak. However, to reproduce the spatiotemporal properties of experimentally-observed large-scale cell flows requires additional interactions between moving cells which align the directions of movement between neighbors. Since our simulation results are agnostic concerning the specific molecular mechanisms for this coordination, we impose the coordination as a general numerical condition rather than as the result of a *specific* cellular or sub-cellular mechanism. In the absence of further experimental data, we demonstrate simply that coordination of *some* type is required and leave the identification of the specific biological mechanisms to future experimental, theoretical and numerical research.

## Results

In this section, we develop simulations, first of primitive streak induction (which creates the initial conditions for primitive streak progression), then of primitive streak progression itself. We identify key possible cell-level mechanisms, especially chemoattraction, chemorepulsion and differential adhesion, from experiments, discuss the simplifications we make to implement them in our simulations, then investigate simulation results for different combinations of these mechanisms and briefly compare to experiment (summarized in Table 1). In all cases, simulation mechanisms and parameters discussed at any stage apply to later-discussed simulations unless we specify otherwise.

**Figure 2:**
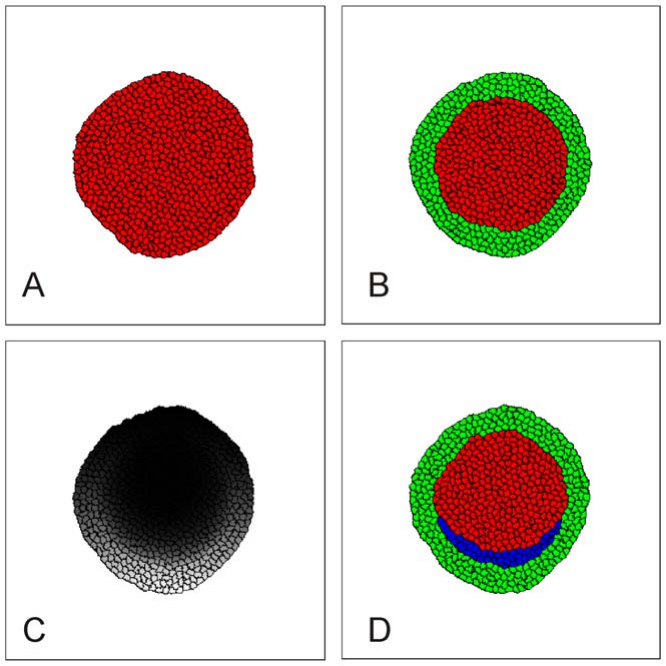
Simulation of differentiation in the early epiblast. The embryo in these simulations contains (initially) 625 cells, *i.e.* 1 simulated cell corresponds to 16 cells in the real embryo. (**A**) Simulation showing that the embryo attains a stable circular shape, provided that the adhesion between cells is strong enough (*J_1,2_>2J_2,2_*). (**B**) To create the AP and AO we assign the AP cell type (red) to all cells whose centre-of-mass lies inside a circle of radius 77 voxels from the center of the embryo and the AO cell type (green) to the remaining cells. The AO (green cells) correspond to the AO in Fig. 1A (area where Wnt8c RNA is expressed). (**C**) Concentration field of the differentiation morphogen in the epiblast (according to Eq. 9). Gray-scale indicates concentration from 0 (black) to 1.5 (white). (**D**) Simulation of mesoderm (blue) differentiation. AP cells differentiate into mesoderm if the concentration of the differentiation morphogen in (C) is greater than 0.7. Blue cells form a Koller's Sickle, *i.e.* corresponding to the Wnt8c RNA expressing area in Fig. 1B. See Supplementary Materials Movie S2 for a movie of this process. See Simulation Details and Supplementary Materials Table S1 for the model architecture and parameter values. Simulations generated using the code in Supplementary Materials Code S1.

The simulations in Figs. 2–6 used the simulation code which we provide in Supplementary Materials Code S1, while the results in Fig. 7 used the CompuCell3D package (http://www.compucell3d.org).

**Figure 3 pone-0010571-g003:**
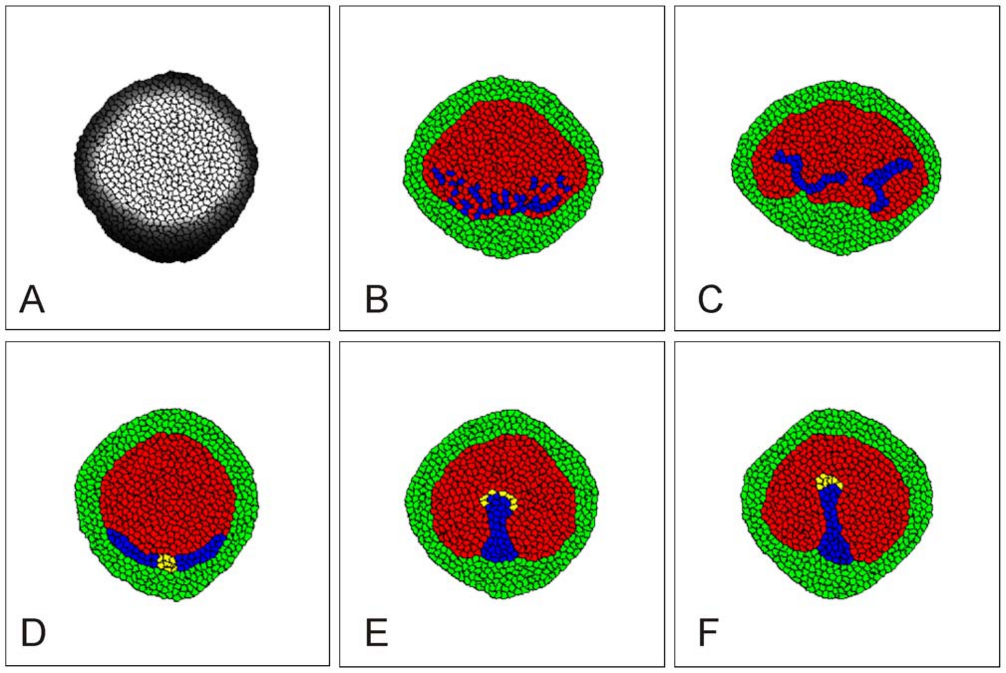
Migration of S cells (blue) and ST cells (yellow) in response to an attractant generated by AP cells (red) (M1). (**A**) Concentration of the chemotactic agent. Gray-scale indicates concentration from 0 (black) to 1.5 (white). (**B**) Typical pattern at 5000 time steps beginning from Fig. 2D, when all AP mesoderm cells respond chemotactically and have the same adhesivity *J_1,3_ = 3* (for remaining values of *J_i,j_* see Eq. 2). The migrating mesoderm cells disperse in the AP. See Supplementary Materials Movie S3 for a movie of this process. (**C**) Typical pattern at 5000 time steps beginning from Fig. 2D, when all AP mesoderm cells respond chemotactically and adhere more strongly to each other than to cells in the AP and AO, *J_1,3_ = 7*, *J_2,3_ = 9*. The migrating sickle cells form streams. (**D, E**) Computational results when only a small, are chemotactically sensitive to the chemo-attractant. (**D**) Initial location of the subgroup of mesoderm cells (yellow) that will ultimately form the streak tip (ST). (**E**) Typical pattern at 7000 time steps beginning from the conditions in D, when only ST mesoderm cells respond chemotactically and the adhesion matrix *J* is that in Eq. 2. See Supplementary Materials Movie S4 for a movie of this process. (**F**) Typical pattern at 7000 time steps beginning from **D**, when only ST mesoderm cells respond chemotactically and the adhesion matrix *J* is that in Eq. 2 except that *J_3,3_ = J_4,4_ = 2* and *J_3,4_ = 4*. Chemotaxis follows Eq. 4, with *β_k_ = 80* if a cell responds chemotactically and *β_k_ = 0* otherwise. See Simulation Details for other parameter values. Simulations generated using the code in Supplementary Materials, Code S1.

**Figure 4 pone-0010571-g004:**
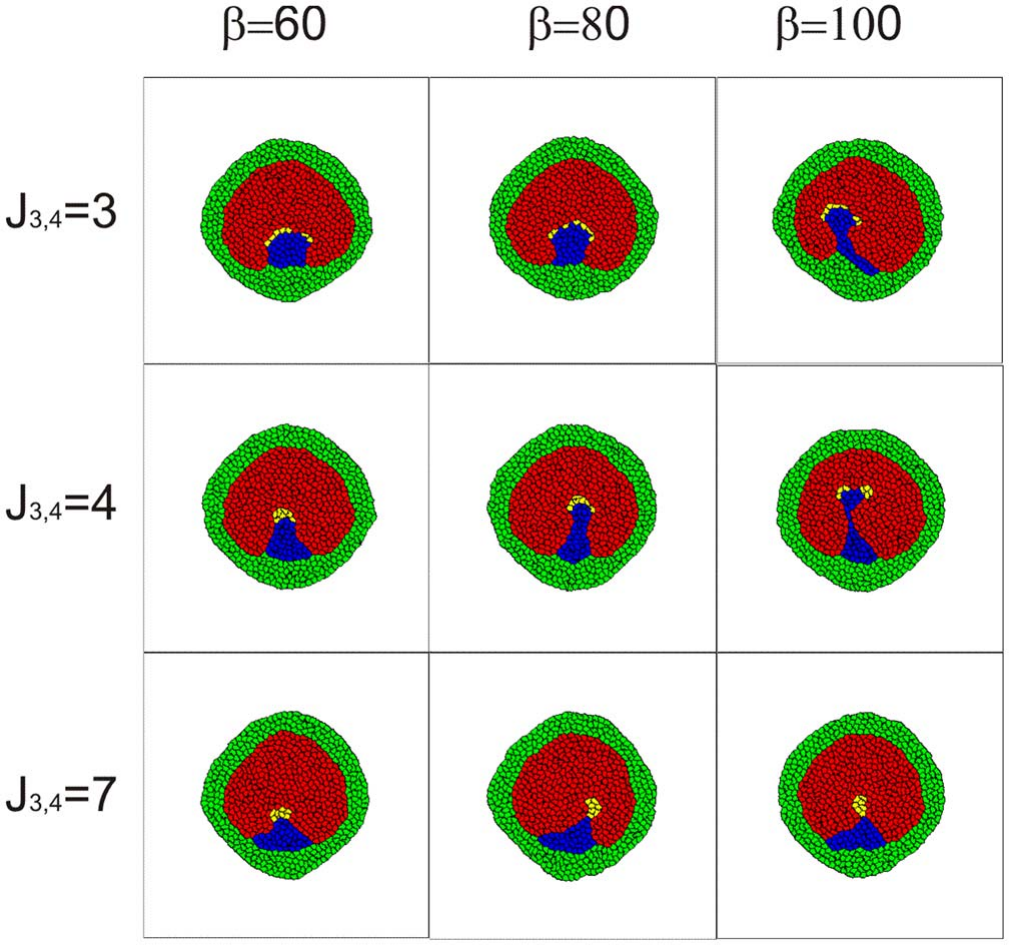
The effects of the strength of the chemotactic response (*β* in Eq. 4) and adhesion between ST (yellow) and S (blue) cells (*J*_3,4_) on the dynamics of the formation of the primitive streak. Results are shown after 7000 simulation time steps starting from the initial condition given in Fig. 3D using mechanism M1. The image in the middle of the panel corresponds to the parameters used in the simulation presented in Fig. 3F.

**Figure 5 pone-0010571-g005:**
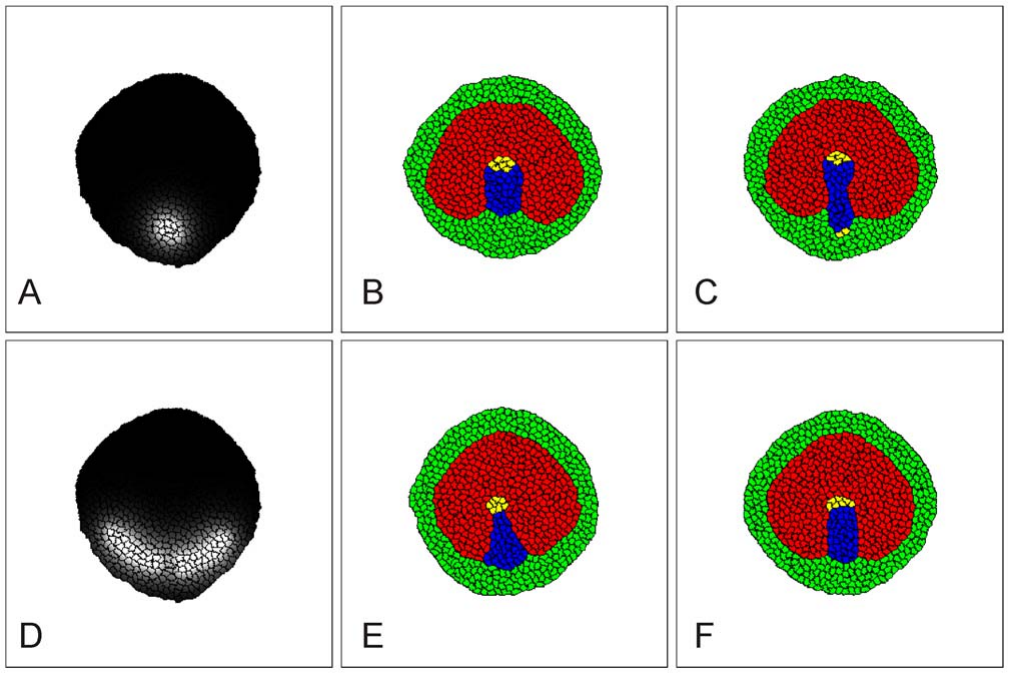
Formation of the primitive streak for different chemotactic mechanisms. (**A**) Typical concentration of a chemotactic agent produced by ST cells (mechanisms M2, M2b and M4). Gray-scale indicates concentration from 0 (black) to 1.5 (white). (**B**) Typical pattern at 7000 time steps beginning from Fig. 3D, when ST cells produce a repellent for AP cells (*β_k_ = −60* for AP cells) (mechanism M2). See Supplementary Materials Movie S5 for a movie of this process. (**C**) Typical pattern at 7000 time steps beginning from Fig. 3D, when ST cells produce a repellent for AP and AO cells (*β_k_ = −60* for AP cells and *β_k_ = −15* for AO cells) (mechanism M2b). (**D**) Typical concentration of a chemotactic agent produced by S cells (mechanisms M1, M3). Gray-scale indicates concentration from 0 (black) to 1.5 (white). (**E**) Typical pattern at 7000 time steps beginning from Fig. 3D, when S cells produce a repellent for ST cells (*β_k_ = −40* for ST cells) (mechanism M3). See Supplementary Materials Movie S7 for a movie of this process. (**F**) Typical pattern at 7000 time steps beginning from Fig. 3D, when ST cells produce an attractant for S cells (*β_k_ = 40* for S cells)(mechanism M4). Chemotaxis follows Eq. 4. with *β_k_ = 0* if a cell does not respond chemotactically. In (A)–(F) *J_3,3_ = J_4,4_ = 2*, *J_3,4_ = 4*, other values as in Eq. 2. In (E) and (F) *J_2,4_ = 9*, other values as in Eq. 2. See Simulation Details for other parameter values. Simulations generated using the code in Supplementary Materials, Code S1.

**Figure 6 pone-0010571-g006:**
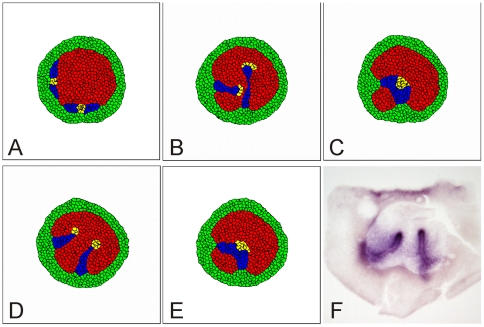
Interaction between two primitive streaks for different model hypotheses. (**A**) Initial cell configuration, with separate groups of S and ST cells at the bottom and the left side of the embryo. Each group can extend to form a primitive streak. (**B**) Typical pattern at 7000 time steps beginning from Fig. 6A, when AP cells produce an attractant for ST cells (mechanism M1). The two extending primitive streaks do not interact until they contact each other (See Supplementary Materials Movie S8, Middle panel). (**C**) Typical pattern at 7000 time steps beginning from Fig. 6A, when ST cells produce a repellent for AP cells (mechanism M2). The primitive streaks attract each other, resulting in collision and fusion of their tips (See Supplementary Materials Movie S8, Right Panel). (**D**) Typical pattern at 7000 time steps beginning from Fig. 6A, when S cells produce a repellent for ST cells (mechanism M3). The extending streaks repel each other, so the tips bend apart (See Supplementary Materials Movie S8, Left panel). (**E**) Typical pattern at 7000 time steps beginning from Fig. 6A, when ST cells produce an attractant for S cells (mechanism M4). The extending streaks fuse after collision. See Supplementary Materials Movie S8 for movie of these processes. (**F**) Experiment showing an embryonic twin with two spontaneous streaks. The extending streaks repel each other so the tips bend apart. Streaks visualized through *in situ* hybridization for expression of Brachyury RNA [25]. See Fig. 5 and Simulation Details for parameter values. Simulations generated using the code in Supplementary Materials, Code S1.

**Figure 7:**
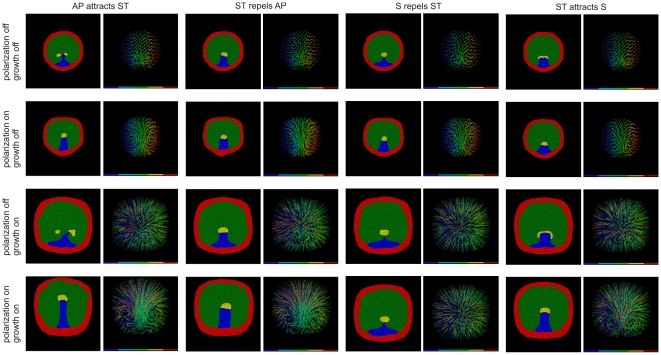
Cell flow patterns during streak formation for different mechanisms. Our four models (M1)–(M4) for cell attraction and repulsion and four models of growth and induced polarization produce sixteen possible sets of combined mechanisms. (**Left**) Typical cell patterns at 7000 time steps beginning from Fig. 3D and (**Right**) Corresponding cell-flow velocity fields for each case. In the absence of proliferation, limited, local vortical motion occurs without induced polarization. However, large-scale vertical motion requires induced polarization. Chemorepulsion mechanisms (mechanism M2, ST repels AP) and (mechanism M3, S repels ST) produce the most robust streak/streak tip structures. Simulations generated using CompuCell3D. For parameters, see Supplementary Materials Table S1.

### Simulation of *mesendoderm* induction

Experiments have shown that streaks can form in isolated pieces of epiblast in the presence of appropriate growth factors, indicating that the epiblast contains all the machinery necessary to produce a streak [22], [23]. Experimentally, the streak forms from cells overlaying and just anterior to Koller's Sickle, which differentiate into mesoderm due to signals (Wnt, BMP) coming from the extra-embryonic ectoderm of the AO. In keeping with these experimental observations, we assume that the PS forms in the epiblast and that only signals originating in the epiblast (both in the AP and AO) affect the PS.

In our simulations of induction, the epiblast consists of a two-dimensional (*2D*) representation of connected, nearly incompressible epithelial cells whose outer ring forms the epithelial part of the AO. Based on the experimental results we discussed in the preceding paragraph, we assume that induction of the sickle-shaped mesoderm depends on an unspecified morphogen, whose kinetics depend on an anterior-posterior asymmetry of the embryo due to gravity and the rotation of the egg in the oviduct [24]. In the absence of detailed molecular data, we propose the following simplifying assumptions: only the AO produces the morphogen controlling mesoderm differentiation and its production rate increases towards the posterior end of the epiblast, resulting in high morphogen concentrations at the posterior end of the AO. We assume that mesoderm differentiation takes place in the AP wherever the concentration of the morphogen exceeds a threshold value.

Under these assumptions, a sickle-shaped mesodermal area forms at the border between the AO and AP as observed in experiments (Fig. 2D and Supplementary Materials Movie S2). This sickle-shaped area of mesendoderm in the circular embryo forms the starting point for all further simulations in this paper.

### Remodeling of the mesendoderm to form the primitive streak

The primitive streak forms when the initially sickle-shaped domain of mesendoderm rearranges into a structure extending along the midline of the embryo in an anterior direction (Fig. 1A, B, D, E, G, H). Simultaneously, large-scale counter-rotating vortex flows develop, which merge at the site of streak formation (Fig. 1C, F, I and Supplementary Materials Movie S1) [4]. We assume that cells in the epiblast chemotax in response to one or more diffusible agents.

#### Initial Chemoattraction Hypothesis

Experiments have not yet established which cells produce which chemotactic agents, which cells respond, and whether their response is attractive, repulsive or both. Therefore, we first investigate the role of chemotactic signaling and response in the morphogenesis leading to the formation of the PS.

Since experiments show that cells move towards the centre of the streak, a logical first assumption would be that cells in the epiblast produce an attractant for mesoderm cells. We start by assuming that AP cells produce a chemotactic agent and that all cells degrade it, so its concentration is maximal at the centre of the epiblast (Fig. 3A). We assume that all mesoderm cells move chemotactically up this gradient (chemoattraction).

#### Differential cell adhesion under the first chemoattraction hypothesis

Experiments show that the epiblast cells adhere to each other through a variety of lateral junctions but do not provide data on the cell-cell adhesivities. Therefore we must consider the effects of differing relative adhesivities (*adhesion hierarchies*) between cell types.

We begin with the null hypothesis that AP mesoderm and AP cells adhere to their own cell type and to the other cell types equally strongly. In this case, the simulated chemotactically-active mesoderm cells disperse into the AP and do not form a streak (Fig. 3B and Supplementary Materials Movie S3).

We therefore assume that the adhesive contacts between mesoderm cells are stronger than those between mesoderm cells and other cells in the epiblast, with an intermediate adhesivity between mesoderm and other cell types. In this case the simulated mesoderm cells remain grouped together as observed experimentally (Fig. 3C). We therefore employ this adhesion hierarchy in all further simulations.

#### Second chemoattraction hypothesis

However, in our simulations with differential adhesion and equal response of all cells to a chemotactic factor produced in the AP, simulated migrating mesoderm cells organize into a few streams which move inward, but fail to form a defined streak (Fig. 3C). We conclude that mesoderm cells cannot all equally respond to a chemotactic factor produced in the AP.

Fate-mapping experiments have shown that the cells in the middle of the sickle-shaped mesoderm later contribute to the tip of the streak, which then transforms into Hensen's Node after the streak has fully extended. The tip cells become recognizable during streak extension because they express tip-specific genes such as Chordin, Hnf3β, cNot1 and Sonic Hedgehog [25].

Therefore, we next assume that only this small group of cells (streak tip or *ST* cells) respond chemotactically (Fig. 3D). This assumption is the limiting case of a more general hypothesis that cells in this group have stronger chemotactic response than more posterior mesoderm cells.

#### Differential cell adhesion under the second chemoattraction hypothesis

In order for simulated posterior streak cells (S) cells to stay in contact with the streak tip (ST) cells and follow them, we also must make the adhesion between S and ST cells stronger than between S cells and other cells in the epiblast. With these modeling assumptions, our simulations show a streak forming along the midline of the embryo (Fig. 3E and Supplementary Materials Movie S4). Further model simulations show that this phenomenon is robust, *i.e.* it is consistent despite variation of the model parameters, although the rate of streak formation and its resulting geometry depend on the adhesive properties of S and ST cells as well as on the strength of the chemotactic movement of ST cells. Fig. 4 shows the shapes of simulated primitive streaks for different adhesive and chemotactic properties of ST cells after 7000 simulation time steps of progression, starting from the condition presented in Fig 3D. Looking at these images we conclude that:

1. An excessively large chemotactic response amplitude (a very large *β_k_* in Eq. 4) results in the breakup of the tip and can even cause the breakup of the primitive streak (see images in the right column of Fig. 4).
2. Reduced chemotactic response amplitude (decreased *β_k_* in Eq. 4) reduces the rate of primitive streak progression. This reduction might be not crucial in comparing simulation outcomes with biological observations as we can make a readjustment by rescaling the simulation's time and space units (see Time and Space units in the Simulation Methodology section). While such rescaling would change the diffusion coefficients and kinetic rates of the chemotactic agents in the simulation, these changes would not exceed a factor of two, within the estimated errors for the values of these constants in experimental conditions. Thus, the rescaled time and space units would be as acceptable as the units we have proposed in the Simulation Methodology Section.
3. Increase in S/ST adhesion (corresponding to smaller *J*_3,4_ and to the first row of images in Fig. 4) causes the streak to extend more slowly and to broaden. ST cells spread widely over the S cells and can even split, breaking the tip in two.
4. Decrease in S/ST adhesion (corresponds to higher *J*_3,4_ and to the third row of images in Fig. 4) causes the actively moving ST cells to peel away from the passive S cells. When the area of ST/T contact shrinks too much, the ST cells stop moving. As a result primitive streak progression stops.

#### Additional chemotaxis hypotheses: Both attraction and repulsion can drive streak extension

We next investigate whether production of attractants or repellents by mesoderm cells can result in streak formation. Our simulations indicate that several scenarios can result in the formation of streaks (See Table 1).

Experimental data on the expression of potential guidance factors do not necessarily show that AP cells secrete an attractant for ST cells. Mesoderm cells express many genes coding for signaling molecules, for example, members of the FGF and VEGF families and scatter factor, that act chemo-repulsively during later stages of chick development [26], [27], [28], [29], [30], while cells in the surrounding epiblast express many receptors, especially for FGFs [31]. In other contexts, some of these factors act as attractants and others act as repellents. The organization of potential signals and the corresponding distribution of receptors could indicate that the mesoderm cells secrete factors that control the behavior of epiblast cells, for instance by repelling them.

Since the key morphogenetic process we investigate involves mesoderm streak (*S*) and streak tip (*ST*) cells moving in a posterior to anterior direction, the most plausible mechanisms involve either the attraction by anterior cells of more posterior cells or the repulsion by posterior cells of anterior cells. How can repulsion form a streak? If the ST cells pushed the AP cells, they could drive the PS forward because of differential cell adhesion and cell incompressibility. If ST cells generate a chemical gradient that causes AP cells nearby to move anteriorly, a region of low pressure develops between the ST and AP cells, causing the ST cells to follow the very AP cells they are pushing away.

We have identified four simple, plausible chemotaxis mechanisms which can explain the progression of the PS: (*M1*) AP cells secrete an attractant for ST cells, (*M2*) ST cells secrete a repellent for AP cells, (*M3*) S cells secrete a repellent for ST cells and (*M4*) ST cells secrete an attractant for S cells.

#### Summary of chemotaxis models and results (see Table 1)

M1. **AP cells attract ST cells**: We have described this mechanism in detail above. It successfully produces streaks (see Fig. 3 and Supplementary Materials Movie S4).

M2. **ST cells repel AP cells**: The streak tip cells produce a diffusible substance which repels epiblast cells in the AP outside the streak. The concentration of the repellent is maximal in the tip, which is initially localized in the posterior epiblast (Fig. 5A). Cells in the AP next to the tip move away from the tip. Consequently the tip cells move anteriorly into the area vacated by the ‘fleeing’ cells, inducing a cell flow along the midline of the embryo (Supplementary Materials Movie S5) similar to that observed in Figs. 3E and 3F.

M2b. **ST cells repel AP and AO cells**: Cells anterior and posterior to the tip cells move away from the tip. The tip splits and follows both moving cell groups, dividing the tip cells into two clusters, one moving anteriorly, the other posteriorly (Fig. 5C).

M3. **S cells repel ST cells**: Posterior mesoderm cells produce a diffusible chemical which repels streak tip cells (Fig. 5D). Since initially posterior mesoderm cells are lateral to the tip cells (Fig. 3D), repulsion of the tip cells moves the tip cells either in an anterior or posterior direction. Usually, the tip splits into two groups of cells moving in opposite directions. However, if we assume weaker adhesion between tip cells and cells in the AO, the tip cells all move anteriorly (Fig. 5E and Supplementary Materials Movie S6).

M4. **ST cells attract S cells**: The tip cells produce a diffusible agent which attracts the posterior mesoderm cells. Posterior mesoderm cells move towards the tip cells, forcing them to move either to the anterior or posterior. If the adhesion between tip cells and cells in the AO is weak, all tip cells move anteriorly (Fig. 5F and Supplementary Materials Movie S7) otherwise the tip splits, and one group of cells moves towards the anterior, while the other moves towards the posterior.

Experimentally, cells in the epiblast move towards the sickle-shaped mesodermal region overlaying Koller's sickle and towards the primitive streak. This movement might suggest that posterior mesoderm cells produce a diffusible substance which attracts other cells in the epiblast. We found that this assumption alone cannot produce a simulation which generates a primitive streak, although in combination with one of the mechanisms above (M1)–(M4), it increases the extension rate of the primitive streak (data not shown).

#### Interactions between two streaks

The simulations we described above show that several choices of secreting and responding cells can result in streak formation consistent with experimental results. In order to identify the most plausible of these mechanisms, we turn to additional experimental data. Induction of extra streaks, for instance through local application of Vg1, would allow detailed experimental study of multiple-streak interactions [32], [33]. In our experiments, the tips of double streaks arising spontaneously always avoid each other (Fig. 6F and Supplementary Materials Movie S8, Left Panel).

We subjected the simulation models above (M1–M4) to the test of reproducing this observation. These tests show the following:

1. If AP cells attract ST cells (M1), the two primitive streaks do not affect each other until they collide and merge (Fig. 6B and Supplementary Materials Movie S8, Middle panel).
2. If ST cells repel AP cells (M2), the primitive streaks attract each other so that their tips fuse (Fig. 6C and Supplementary Materials Movie S8, Right panel).
3. If S cells repel ST cells (M3), the extending streaks repel each other (Fig. 6D and Supplementary Materials Movie S8, Left panel).
4. If ST cells attract S cells (M4), the extending streaks do not affect each other until they collide and fuse (Fig. 6E).

Thus our simulations support hypothesis (M3), posterior mesoderm cells produce a diffusible chemical which repels streak tip cells.

### Cell polarization and velocity alignment between neighboring cells result in large-scale tissue flows

The experimental data in Figs. 1 C, F, I show that simultaneous with streak initiation, all cells in the epiblast begin to move and organize into two vortices touching along the primitive streak. We have calculated cell flow-velocity profiles in our simulations to compare with experiment. Although many of the simulations we described above form primitive streaks, their velocity profiles differ from those in experiments and the vortices form slowly as the streak progresses (see the first row of images in Fig. 7 and Table 1).

Three aspects of the velocity and vorticity field strengths and time dependencies observed in real embryos suggest that an additional mechanism is significant during primitive streak progression:

1. The vortices develop concurrently with streak formation.
2. The vortices are well-coordinated and span the entire mesoderm.
3. The maximum velocity of cells in the vortices is greater than that of the progressing primitive streak.

These behaviors require a mechanism that results in rapid and strong local co-alignment of the velocity vectors of neighboring cells in the epiblast. While pressure interactions between neighboring cells do produce a gradual and partial co-alignment of velocities, this mechanism is too weak and slow to explain the experimentally-observed spatiotemporal behavior of the vortices. A few of the many possible biological and physical mechanisms which could induce local co-alignment of cells' in-plane velocities include: (1) mechanical adhesion between the epiblast cells due to their tight junctions, which could make the epiblast behave like a viscoplastic material, (2) weaker adhesion between the cells, which could make the epiblast behave like a highly viscous fluid, (3) cell-cell contacts via desmosomes which could cause mechanical alignment of the in-plane polarity of neighboring cells, (4) strain-induced alignment of the ECM, which could guide cell motion, (5) Wnt-PCP interactions (chemical signaling) and (6) moving in-plane polarized cells could emit a short-range attractant from their posterior ends, as in *Dictyostelium* aggregation [34]. (7) Additional mechanical interactions are also possible. When cells pull up their trailing end, they both pull on the basement membrane through integrins and on neighboring cells through cadherins. Either interaction could polarize neighboring cells, resulting in coalignment of movement; *e.g.*, fibroblasts on elastic substrates move to regions of higher tension [35], [36].

Since all these, and a variety of other potential mechanisms, would produce mathematically identical effects, our simulations cannot determine the mechanism producing the induced polarization at this point.

Since we do not know which biological mechanisms are significant, we implement local co-alignment numerically through an abstract *polarization vector* that influences a cell's velocity. This vector need not be equivalent to actual planar cell polarization. Mathematically, simulating chemotactic response using this polarization vector is similar to the method we used for pure chemotaxis. The combination of chemotaxis and local co-alignment of velocity creates highly coordinated large-scale vortex flows (see the second and fourth row images in Fig. 7) similar to those observed in experiments (Figs. 1C, F, I). In addition, the vortices form rapidly after the initiation of streak extension.

### Cell proliferation

None of our simulations so far included cell division, which occurs at a low, apparently roughly uniform rate throughout the AP during gastrulation. Since the growth and proliferation of cells affects both their local and bulk motion, we checked the effect of such diffuse proliferation on our simulations. In our simulations cell division increases cells' outward radial velocity but does not produce vortices (see the third row of images in Fig. 7). Proliferation also contributes to cell mixing and small-scale intercalation, as indicated by the color coding of the cell tracks from left to right. The overall effect of proliferation on streak progression is to weaken the vortices, but at experimentally-realistic rates of cell division, the disruption should not be significant. Similarly, while we have not conducted explicit simulations, we would expect the ongoing loss of cells throughout the AP to ingression to have minimal effect on progression and vortex formation.

## Discussion

### Chemotaxis

Based on experimental [4], [11] and theoretical [16] considerations, we assumed that streak formation involves chemotaxis of cells in the epiblast. This paper used the GGHM [18] to investigate a number of different hypotheses concerning the mechanisms transforming the initially sickle-shaped domain of mesoderm cells into a structure extending along the midline of the embryo and creating simultaneous large-scale vortical cell flows. Table 1 summarizes our results concerning the formation of the streak (Figs. 3–7), while Supplementary Materials Table S1 provides the simulation parameters we used to obtain these results.

Surprisingly, a streak can form both as a result of chemo-attraction and chemo-repulsion. The four chemotactic mechanisms we investigated form two groups, where exchanging both attraction with repulsion and signaling and responding cell populations produces the same effect. In the first group, AP cells produce a signal which attracts ST cells (M1), or ST cells produce a signal which repels AP cells (M2). In the second group, ST cells produce a signal which attracts S cells (M4), or S cells produce a signal which repels ST cells (M3). All these mechanisms form streaks which look roughly similar. However, when we compare the interaction between two primitive streaks simultaneously progressing in a single embryo, these mechanisms predict different outcomes. Preliminary observations from our own experiments (Fig. 6F) and those published by others, indicate that the tips of streak never fuse, favoring (M3), where the streak cells (S) produce a signal which repels the tip cells (ST).

The FGF family of growth factors, especially FGF8, which is expressed in the streak but not at the very tip [26], [37], are good candidate repellents for streak cells, since our prior experiments have shown that FGF8 repels mesoderm cells during their movement away from the primitive streak after their ingression and since FGF receptors are expressed widely in the epiblast [15], [31]. So far, experiments have failed to distinguish FGFs' roles as directors of cell differentiation into mesoderm from their roles in cell guidance [38]. Other unknown signaling molecules might also perform these functions.

### Long-range coordination of cell movement as a consequence of local induced polarization

**Figure 8:**
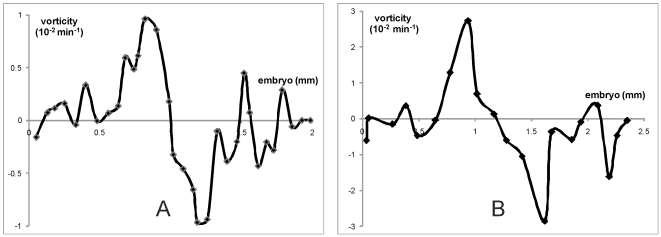
Simulated vorticity of cell flows measured along a horizontal line, perpendicular to the primitive streak and crossing the center of the embryo. (**A**) From a simulation where S cells repel ST cells (mechanism M2), polarization off, growth off (see Fig. 7). (**B**) From a simulation where S cells repel ST cells (mechanism M2), polarization on, growth on (see Fig. 7). Both plots are rescaled to units of mm (to measure the distance along the measurement line) and min^−1^ (to measure vorticity) according to the space and time unit definitions given in the Simulation Methodology Section. The midline of the embryo crosses the plots in the middle (1 mm in A and 1.25 mm in B). The vorticity is measured according to the formula 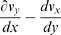 where 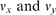 are horizontal and vertical components of cell flow velocities. These velocities calculated as the ratio of total cell shifts over all simulation time. The vorticity is negative for clockwise and positive for counter-clockwise rotation. It is zero at the midline of embryo and increases to the left and decreases to the right with maximum/minimum at about quarter of embryo's radius from the midline. It returns smoothly to zero at the embryo boundaries.

Chemotactic movement in response to local signals forms a streak through small local cell rearrangements, with little movement far from the streak (Fig. 1 and Fig. 7). To obtain large-scale flows requires some local mechanism that aligns the movement directions of neighboring cells, *i.e.*, the tissue has to have viscous or plastic effective properties [12]. Our prior experiments have shown that all cells must move actively [4], *i.e.* cells are not just passively pulled along by other cells. In order to obtain large-scale cell flows in simulations we had to include co-alignment interactions between moving cells. We introduced induced polarization, where we assume that the direction of movement of a given cell depends not only on its own response to chemo-attractants and repellents, but on neighboring cells. Our simulations have shown that a combination of chemotaxis and induced cell polarization reproduces the experimentally-observed large-scale cell flow patterns. The vortex flows primarily arise from recirculation to replace cells driven by chemotaxis, with the size of the vortex increasing with the degree of cell-cell co-alignment. The effect of induced polarization on the formation of global cell flows over the epiblast can be quantified by the measurement of the vorticity of the cell flows. These measurements show that induced polarization significantly increases the vorticity of cell flows in our simulations (Fig. 8).

Together, our simulations show that chemo-attraction and chemo-repulsion are strong candidate mechanisms for the guidance of cell movement during streak formation, provide valuable insight into the potential locations of attractant/repellent production and response, and suggest experiments to identify candidate attractants/repellents. We can easily extend our simulations to predict experimental flows in response to ectopic attractants/repellents and thus distinguish among our hypothetical mechanisms (M1)–(M4) and other possible mechanisms. Our simulations also require the streak cells to adhere more strongly to each other than to other cells in the epiblast for them to remain grouped together, an important testable prediction, which should inspire experimental verification.

## Methods

Despite a large body of experimental data on patterns of differentiation, cell division, and movement, we are still learning how embryos integrate and control these processes in gastrulation. Biological experiments on living cells frequently cannot isolate individual mechanisms. Realistic computer simulations provide an alternative method for screening hypothetical mechanisms. In this paper have we treated our simulations as *in-silico* experiments to evaluate potential mechanisms for PS formation and vortex motion.

We perform these simulations using the multi-cell GGHM. The GGHM simulates cells as autonomous, spatially-extended agents living in a computer lattice that (1) individually control their intrinsic properties such as volume, secretions, polarity, *etc.* and (2) interact with other cells in realistic manners via specified rules. A GGHM implementation describes a biological cell as a contiguous, irregularly-shaped set of voxels in a lattice. Specific rules allow cells to move and change shape by reassigning different voxels to different cells. The implementation stores the concentrations of chemicals in parallel lattices. When cells secrete chemicals, the GGHM updates the concentration fields, which evolve using numerical schemes for solving the specified diffusion equations (Eq. 10).

### Mathematical and Numerical Details

GGHM cells are 2D patches of voxels on a lattice. We initialize the embryo as a disk of cells (see Fig. 2A). A ring of AO cells approximately three cells wide forms the perimeter of the disk. AP cells fill the area inside this ring. Cells move according to random fluctuations which represent cytoskeletally-driven cell motility and a description of all cell interactions as an *effective energy*, *E*. We repeatedly select a random source voxel, randomly select a neighboring target voxel and calculate how the effective energy would change if the source voxel displaced the target voxel. If this change decreases the effective energy, we allow the change to occur; if the effective energy would increase, we make the change with a probability *p*, which decreases according to a Boltzmann factor:
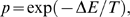
(1)where the parameter *T* represents the intrinsic motility of the cells (we set *T = 6* in all our simulations). The effective energy includes the adhesive contacts between cells, cell size, chemotactic response and cell polarization as follows:

1. Interactions between neighboring voxels have an effective energy *J_k_*_,*l*_ (*J_k,l_ = J_l,k_*) if they belong to different cells (*k* and *l* represent the types of these cells) or 0 if they belong to the same cell. *J_k_*_,*l*_ characterizes the strength of a cell's adhesive contacts (stronger contacts correspond to smaller *J*). We represent the substrate as a special type of *generalized cell*. Our simulations used up to 5 generalized cell types:
  Cell type 1 – the underlying substrate (*Sub*)
  Cell type 2 – cells which form the *Area Pellucida* (AP)
  Cell type 3 – cells which form the *Area Opaca* (AO)
  Cell type 4 – posterior streak cells (S)
  Cell type 5 – streak tip cells (ST)
  The default adhesion matrix was:
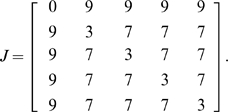
(2)Cells adhere more to each other than to the surrounding area (*J_k,l_ = 7* when *k*,*l*>0 and *J_k,0_ = 9* for *k*>0), that is, the adhesion forces the cells to stay in one group (not to disperse) to form a tissue. Moreover, cells of the same cell type are more adhesive to each other (*J_k,k_ = 3*, *k*>0) than to the cells of other types (*J_k,l_ = 7* when *k*≠l), allowing for cells of the same type to stay in compact groups (stay sorted) as is observed in experiments.
2. We control the volume of each cell, *V_k_*(*t*), using a target volume, *T_k_*. Cell *k* has a *volume effective energy*:
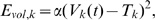
(3)where *α* is a positive constant. To allow growth and proliferation of cells, *T_k_* may vary in time.
3. To implement cell movement in response to a chemotactic agent with concentration *u*, we define a *chemotactic effective energy*:
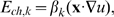
(4)where *β_k_* is a constant describing the chemotactic response of the *k* ^th^ cell to the chemotactic agent *u* and ***x*** is a vector representing the local displacement of the cell's boundary. Relocation of the cell's boundary changes the chemotactic effective energy depending on the local gradient of the chemotactic agent. A positive *β_k_* produces chemo-repulsion while a negative *β_k_* produces chemo-attraction. This form of chemotaxis in the GGHM corresponds to the standard Keller-Segel chemotactic flux in PDE models.
4. An alternative interpretation of the chemotactic response of the cells considers cell polarization. We can rewrite the chemotactic effective energy (Eq. 4) as a *polarization effective energy*:
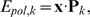
(5)where the vector:
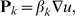
(6)represents the polarization of cell *k*. This rewriting does not affect the behavior of the tissue, but it does allow introduction of additional interactions between polarized cells. To simulate the effect of moving cells on their neighbors so that the neighbors also move we include induced polarization following an ordinary differential equation typical for flock models of orientation:
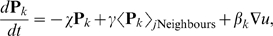
(7)where the first term on the RHS represents a cell's decaying memory of its previous polarization, the second term represents the influence of the average polarization of the neighboring cells and the last term represents the polarization due to the chemotactic agent. We approximate this ODE by updating the polarization of a cell at each time step according to the polarization of its neighbors:
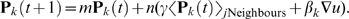
(8)The polarization vector affects a cell's direction of motion by increasing the probability of accepting prospective moves which align it more closely with the direction of polarization.
5. The concentration field of the morphogen responsible for the differentiation of the mesoderm cells obeys the Poisson equation:
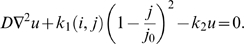
(9)The term *k*_1_(*i*,*j*)(1−*j/j*_0_)^2^ defines the production of the morphogen: the parameter *k*_1_(*i*,*j*) = 1.5×10^−3^ if the lattice site (*i*,*j*) belongs to an AO cell and 0 otherwise. The factor (1−*j*/*j*_0_)^2^ describes the embryo's anterior-posterior anisotropy. *j*_0_ is the vertical coordinate of the most anterior point of the epiblast. The maximum morphogen production rate, *k_1_/k_2_ = 1.5*, occurs at the most posterior point of the epiblast. *k_2_ = 10^−3^* is the decay rate of the morphogen, which is constant over the epiblast. *D = 1* is the diffusion constant of the morphogen. AP cells which sense a sufficiently high level of *u* (here *u*>0.7) differentiate into mesoderm cells.
6. The concentration fields of the chemotactic agents obey a diffusion equation:
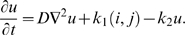
(10)Here, *k_1_(i,j) = 1.5×10^−3^* if the lattice site (*i*,*j*) belongs to a cell which produces the chemotactic agent and 0 otherwise, *k_2_ = 10^−3^*, *D = 1*. The chemical-field lattices have the same size and discretization as the cell lattice.
7. In simulations without cell proliferation, all cells have a constant target volume, *T_k_ = 50*. We set the cell compressibility *α = 0.6*, which is small enough to allow rapid movement of cells but large enough to maintain cell size. In simulations with cell proliferation, we randomly assign initial target volumes to cells in the range 30–70 voxels, then increase the target volume of each cell by one voxel every 20 time steps. When the actual volume of a cell reaches 100 voxels, the cell divides along a randomly oriented line through the cell's centre of mass. After division, the target volumes of both daughter cells reset to 50 voxels, after which they grow as before.

### Time and Space Units

At laying, the embryo contains about 10^4^ cells and is roughly 2 mm in diameter, so the diameter of a cell is about 20 µm. To reduce computation time, our simulations in Figs. 3–7 represent an embryo as consisting of only 625 cells. Thus the diameter of our simulated cells is 80 µm, with each simulated cell representing 16 real cells. Since each simulated cell contains approximately 50 voxels, the voxel size in these simulations is roughly 10×10 µm^2^. and the simulation time step is approximately 3 seconds since primitive streak extension takes 6 hours in experiments and 7000 time steps in our simulations. The diffusion constant *D = 1* thus corresponds to about 10^−7^ cm^2^/sec. We set the relaxation time for the morphogen ODE to *τ* = 10^3^ simulation time units, which corresponds to *3×10^3^* sec and a diffusion length of 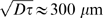, which is roughly 15 cell (or two “computational” cell) diameters. This diffusion length could provide a clue to identifying the chemotactically active cells in experiments.

### Positional Stability of the Embryo

A specialized group of cells on the outer periphery of the vitelline membrane anchor the embryo and keep it under tension. The deeper cells of the AO directly contact the yolk and may also help anchor the embryo. In our simulations, these rigid boundary conditions are essential to holding embryos stationary in space as cells move and, in particular, allowing primitive streak advance and formation of vortices. We employed two types of boundary condition in our simulations. The simulations in Figs. 2–6 did not fix the boundary and calculated all cell displacements and chemical concentrations relative to the embryo's center-of-mass updated every 50 time steps (*i.e.* every 50 time steps, we calculated the embryo's center-of-mass and, if necessary, shifted all calculated lattices to keep them fixed relative to the center-of-mass). In Fig. 7 we assumed that the AO cells secrete an ECM (implemented as a diffusion field) to which they show strong haptotaxis (implemented as a chemotaxis term in the effective energy). The ECM obeys the diffusion equation (Eq. 10) with a very small diffusion constant and decay rate, which leads to a sharp gradient of the ECM field at the embryo boundary that attracts the AO cells, preventing bulk movement of the embryo or rapid shape changes, though AO cells can still move and the AO can gradually change shape.

## Supporting Information

Table S1 Parameters related to diffusion and time scales in CompuCell3D simulations (Fig. 7).
(0.06 MB DOC)

Movie S1 Experimental streak formation and velocity vector field. See Fig. 1.
(7.38 MB AVI)

Movie S2 Mesoderm differentiation above Koller's sickle. See Fig. 2 and text for details.
(3.20 MB AVI)

Movie S3 Dispersion of sickle cells when they all are attracted by AP cells (see Fig. 3B).
(7.73 MB AVI)

Movie S4 Formation of the primitive streak via mechanism M1: AP cells attract ST cells (see Fig. 3E).
(8.43 MB AVI)

Movie S5 Formation of the primitive streak via mechanism M2: ST cells repel AP cells (see Fig. 5B).
(7.58 MB AVI)

Movie S6 Formation of the primitive streak via mechanism M3: S cells repel ST cells (see Figs. 5D and 5E).
(5.73 MB AVI)

Movie S7 Formation of the primitive streak via mechanism M4: ST cells attract S cells (see Fig. 5F).
(10.67 MB AVI)

Movie S8 Interaction of two extending streaks: left panel - streak repulsion via mechanism M3 (Fig. 6D); middle panel - no interaction via mechanism M1 (Fig. 6B); right panel - streaks attract each other via mechanism M2 (Fig. 6C).
(8.45 MB MPG)

Code S1 Archived file containing readme.txt, executable file, and Visual C++ source code for reproducing results presented in Figures 2–6.
(9.31 MB ZIP)
