# Supplementary material for: Modeling Gastrulation in the Chick Embryo: Formation of the Primitive Streak
Source: PLoS One. 2010 May 11;5(5):e10571. doi: 10.1371/journal.pone.0010571 (PMC2868022; doi:10.1371/journal.pone.0010571)
Supplement: Table S1 — Parameters related to diffusion and time scales in CompuCell3D simulations (Fig. 7). (0.06 MB DOC) [file pone.0010571.s001.doc]

| **Physical Parameter** | | **Symbol** | **Physical Value** | **Simulation Value** | **Discussion** |
| --- | --- | --- | --- | --- | --- |
| Time Step | | *TS* | 3 s | 1 | Primitive streak extension takes ~ 6 hours in experiments and 7000 time steps in our simulations |
| Voxel Size | | *l*_v_ | 0.7 to 1.3 μm | 1 | For an embryo ~ 1 mm in diameter, a 12,000-cell 2D simulation with cells with an area of ~ 50 voxels has a diameter of ~ 800 *l*_v_ ⇒ *l*_v_ ~ 1.3 μm. For a simulation with 45,000 cells, *l*_v_ ~ 0.7 μm. |
| Diffusion Constant | | *D* | 10^-7^ cm^2^ s^-1^ (estimated) | 0.14 *l*_v_^2^ *TS*^-1^ | We assume diffusion to be fast, so we neglect advection. The observed physical diffusion constant combines diffusion within cells, diffusion between cells, and diffusion through ECM. |
| Fractional Decay Constant | | *α* | Unknown | 0.0001 *TS*^-1^ | Biologically, decay can result from metabolic uptake, fixation in ECM, chemical instability, *etc*. CompuCell3D multiplies the Field concentration by one minus the constant once each time step. |
| Secretion Constant | | *β* | Unknown | 0.01 *TS*^-1^ | In our simulations one morphogen is secreted. One cell type secretes the morphogen. The secretion rate is constant and the same in all simulations. |
| Morphogen diffusion length (derived quantity: *l*_d_ = (*D*/*α*)^0.5^ | | *l*_d_ | Unknown, but measurable | ~ 37 *l*_v_ | The *diffusion length* characterizes the effective range of a diffusive signal. In our simulations, it also helps define the time scale. |
| Number of Cells | | *N* | Roughly 45000 | 1200 or 45000 | Our coarse-grained simulations used 1200 cells to represent the actual 45,000, so one simulated cell represents 16 real cells Others used the full 45,000. |
| Length Scale | Embryo |  | ~1 mm | ~1000 *l*_v_ | Based on an embryo with 45,000 cells. |
|  | Cell |  | ~20 μm | 7 *l*_v_ |  |
