# Supplementary figures and images for: Modeling Gastrulation in the Chick Embryo: Formation of the Primitive Streak
Source: PLoS One. 2010 May 11;5(5):e10571. doi: 10.1371/journal.pone.0010571 (PMC2868022; doi:10.1371/journal.pone.0010571)

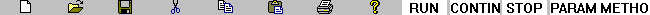

Supplement: Code S1 — Archived file containing readme.txt, executable file, and Visual C++ source code for reproducing results presented in Figures 2– 6. (9.31 MB ZIP) [file pone.0010571.s010.zip › potts3/res/Toolbar.bmp]
